# How Drawing to Distract Improves Mood in Children

**DOI:** 10.3389/fpsyg.2021.622927

**Published:** 2021-02-02

**Authors:** Jennifer E. Drake

**Affiliations:** Department of Psychology, Brooklyn College and the Graduate Center, City University of New York, Brooklyn, NY, United States

**Keywords:** drawing, emotion regulation, distraction, absorption, imagination

## Abstract

Previous research has shown that drawing improves short-term mood in children when used to distract from rather than express negative thoughts and feelings. The current study sought to examine (a) *how* drawing might elevate mood in children ages 6–12 by examining the role played by absorption, enjoyment, and perceived competence as well as entering an imaginary world; and (b) whether children spontaneously use drawing to distract from a sad mood. Across three studies, children were asked to think of a disappointing event. After a sad mood induction, they drew for 5 min. Mood was measured before and after the mood induction and after drawing. Three main findings emerged. First, drawing to distract led to greater absorption and enjoyment than did drawing to express. Second, children’s mood improved equally when drawing imaginary and real scenes showing that the key ingredient is that the content of the drawings be distracting in nature. Third, drawing improved mood even when children were given no instructions on the content of their drawings and children were more likely to use drawing as a way to distract themselves from a sad mood. These studies help to define the characteristics of drawing activities that foster mood improvement in children and highlight the important role of the arts in emotion regulation.

## Introduction

Whether sadness over a lost pet, exuberance over winning a game, or fear when confronted by a bully, children encounter emotionally arousing situations on a daily basis (Denham, 1998). With development, children learn to regulate their emotions by monitoring, evaluating, and modifying them (Thompson, 1994). An important aspect of emotion regulation is learning which strategies to use when confronted with an emotionally arousing, stressful situation.

Children use a variety of strategies to regulate their emotions (e.g., problem-solving, comfort seeking, distraction, escape, and information seeking) with the most common emotion regulation strategy being distraction (Skinner and Zimmer-Gembeck, 2007). While distracting themselves, children are not actively avoiding their feelings, but rather are focusing on something else to help them adjust their feelings. Children regulate their emotions primarily through behavioral distraction (e.g., playing games) but also through cognitive distraction (e.g., thinking about something fun). Behavior distraction emerges in the preschool years whereas cognitive distraction emerges in early childhood. It is not until children reach early adolescence that they can seek out and plan their own distracting activity (Zimmer-Gembeck and Skinner, 2011). With development, children come to recognize that behavioral distraction is effective when it involves an absorbing activity that displaces negative thoughts with positive thoughts (Harris, 1989). An example of one such pleasurable activity may be drawing.

Children are natural artists and, when given the opportunity, most are highly absorbed in the act of drawing from a very young age (Gardner and Winner, 1982; Jolley et al., 2000). When drawing, children are engrossed, focused, engaged, and playful. It seems likely then that engaging in this activity would help them regulate their emotions. Children have been found to gravitate to the arts during emotionally troubling times. For example, children used drawing as a way to cope with and understand hurricane Katrina (Dewan, 2007). In clinical settings, art therapy has been shown to improve well-being and psychological outcomes in children (e.g., Pifalo, 2006; Briks, 2007). Given that children are drawn to art-making, and given the emotional benefits of drawing, it is surprising that very few studies have examined the benefits of everyday drawing in non-clinical settings.

It has been established that drawing can improve mood in children ages 6–12 (Drake and Winner, 2013). Drake and Winner (2013) induced a sad mood by asking children to think of a time when they were really disappointed about something. Children rated how they were feeling after thinking about the event and were randomly assigned either to make a drawing that expressed how they were feeling (by drawing the event that they had thought of) or to make one that distracted them from thinking about what they were feeling (by drawing a house – something unrelated to what had disappointed them). Children drew for five minutes and then rated their mood again. Whereas both groups improved in mood, those in the distract condition improved significantly more than those in the express condition. Those in the distract condition also improved more than a control condition asked to copy a line drawing. The benefits of drawing were greater for younger than older children. Thus, the act of freely constructing an image seems to play an important role in mood improvement.

Does the valence of children’s drawings when used to distract affect mood improvement? One study compared the benefits of drawing to express versus two different drawing to distract conditions – drawing something positive and a free draw condition in 7 and 10 year olds (Brechet et al., 2020). Those in all three drawing conditions improved in mood, but mood improvement was greatest for the draw something positive and free draw conditions. Similar to Drake and Winner (2013), mood improved more for younger than older children. Thus, it did not matter whether children were asked to think about something that made them happy and draw it (draw something positive condition) or were given the choice in determining the content of their drawings (free draw condition). Both distracting drawing activities led to greater mood improvement than the draw to express condition.

Another study examined the benefits of recalling versus drawing an autobiographical memory that was either positive or neutral in valence (Monnier et al., 2016). Recalling a positive autobiographical memory has been shown to be an effective and efficient way to induce a positive mood (Strack et al., 1985). However, as pointed out by Monnier et al. (2016), children may find it difficult to recall detailed information to an experimenter especially for children with delayed language skills. Drawing is a simple tool that children can use to depict their thoughts and feelings in a non-verbal manner. A drawing distraction task may be more effective in elevating mood than a verbal distraction task because drawing not only distracts but also provides pleasure from creating something (White, 1959). Monnier et al. (2016) reported that drawing a memory was just as effective in improving mood as was recalling a memory. However, children reported feeling calmer after drawing a memory than after simply recalling a memory. Furthermore, children who drew or recalled a positive memory had greater mood improvement than did children who drew or recalled a neutral memory. Thus, in the case of drawing something personal, valence matters: positive content that distracts is more powerful than neutral content that distracts.

Four main findings have emerged from the initial work on the benefits of drawing for improving children’s mood. First, drawing to distract is more effective in improving mood in children than drawing to express, but only when children generate the image on their own rather than copy one (Drake and Winner, 2013). Second, no difference in mood improvement is shown when children are prompted to draw a specific scene versus draw whatever they want (Brechet et al., 2020). Third, children report feeling calmer after drawing a remembered event than after simply remembering an event without drawing. Finally, and not surprisingly, the valence of that personal memory matters: children showed greater mood improvement after drawing or recalling a positive memory than a neutral memory (Monnier et al., 2016).

While these studies show clearly that drawing to distract is an effective way to improve children’s mood, there is much that we do not know. What is the role of absorption, enjoyment, perceived competence, and imagination in mood improvement when children draw to distract themselves? And if drawing to distract is such an effective strategy, do children realize this in some way and spontaneoulsy use drawing to distract rather than express? The current set of studies delves deeper and more systematically into the question of *how* drawing to distract elevates mood in children ages 6–12.

It is possible that drawing to distract improves mood in children because it is an absorbing activity that children enjoy and feel competent doing. Work with adults has shown that drawing to distract leads to greater states of flow (an indicator of absorption) than drawing to express (Forkosh and Drake, 2017). Therefore, when children use drawing to distract they might be better able to shift their attention away from what upsets them because they are highly engaged in the activity. Drake and Winner (2013) found that children who used drawing to distract reported greater enjoyment and perceived competence about their drawing experience than children who used drawing to express. Children’s enjoyment and perceived competence when drawing might also be impacted by the children’s age. Younger children report higher levels of enjoyment and perceived competence when drawing than do older children (Drake and Winner, 2013). Even though children’s drawing skills improve with age, children become highly critical of their drawings as they age and as a result, enjoyment and perceived competence when drawing declines with age (Jolley, 2010). Higher perceived competence reported by younger children is not exclusive to drawing. In a similar study, Brechet et al. (2020) found that after a drawing task, younger children rated their overall perceived competence as higher than older children. Study 1 examined potential developmental differences in the role of absorption, enjoyment, and perceived competence when drawing to distract.

Another possible explanation for why drawing to distract might improve mood in children is that it allows children to enter an imaginary world. For example, having an imaginary companion has been found to be effective for dealing with anxiety (Taylor et al., 2011). Engaging in imaginary activities like pretend play or drama has been associated with increases in empathy and the use of adaptive emotion regulation strategies (Hoffman and Russ, 2012; Goldstein et al., 2013; Goldstein and Lerner, 2018). Pretend play offers children a unique opportunity to create and engage in arousing emotional situations where they can express and negotiate their emotions (Bretherton, 1989). Furthermore, when children use their imagination to create an imaginary world, their emotions are directed at the events in that imaginary world rather than at ongoing, actual events (Harris, 2000). Thus, children may be able to effectively shift their attention away from what is upsetting them. Since children often engage in behavioral distraction to regulate their emotions, it seems plausible that creating an imaginary world through drawing might improve mood in children. Study 2 examined whether drawing a scene depicting an imaginary scene leads to greater mood improvement than either drawing a scene depicting a real-life scene or drawing to express.

Given the benefits of drawing to distract (as reviewed above), do children spontaneously use drawing to distract themselves from negative feelings? It seems likely that children would use drawing to distract from negative feelings since distraction is one of the most common emotion regulation strategies used by children (Skinner and Zimmer-Gembeck, 2007). One study with a slightly different age group than the current study (7 and 10 year olds versus 6–8 and 10–12 year olds) found that the majority (89%) of children did use drawing as a form of distraction in a free draw condition (Brechet et al., 2020). This study also analyzed the themes of children’s drawings and found that the content of children’s drawings differed by age: 7 year olds tended to draw familiar environments or people while 10 year olds tended to draw celebrations or leisure activities. Study 3 sought to expand on the work by Brechet et al. (2020) to examine whether children spontaneously use drawing to distract rather than express as well as the reason *why* children choose to draw a particular theme. Do they choose to draw what they are already good at drawing? Do they choose to draw something that had brought them joy? Did they choose to draw something that they observed in their environment? In addition to examining the content of children’s drawings, this study examined what they were thinking about while drawing. This allowed for the assessment of whether the activity served as a form of distraction or expression and whether children’s thoughts were focused on drawing or some other activity.

Across all three studies, it was hypothesized that drawing would improve mood more when used as a form of distraction rather than expression. Study 1 tests the hypothesis that level of absorption, enjoyment, and/or feelings of perceived competence affect mood improvement when using drawing to distract. Study 2 tests the hypothesis that drawing from imagination leads to greater mood improvement than drawing something from real life. Study 3 tests the hypothesis that children spontaneously use drawing as a form of distraction and that while drawing their thoughts are focused on the activity.

It was also hypothesized that younger children (ages 6–8) would benefit more from drawing to distract than older children (ages 10–12). As reviewed above, these two age groups differ in their experience drawing with younger children more absorbed in the act of drawing and older children more critical of their drawings (Gardner and Winner, 1982; Jolley et al., 2000). These two age groups also differ in their use of distraction as an emotion regulation strategy. Older children are more likely to use cognitive distraction strategies (e.g., thinking about something fun) to regulate their emotions. Whereas younger children might be able to use cognitive distraction strategies to regulate their emotions, they more often rely on behavioral distraction strategies (Zimmer-Gembeck and Skinner, 2011). Younger children might show greater mood improvement after drawing because it is a form of behavioral distraction. This is consistent with previous research that has shown drawing to distract improves mood more in younger than older children (Drake and Winner, 2013; Brechet et al., 2020). Thus, the inclusion of these two age groups allowed for the examination of whether there are developmental differences in *how* drawing to distract might improve mood in children.

## Study 1

### Methods

#### Participants

Children (*n* = 130) between the ages of 6 and 12 were recruited from local science and children’s museums. Parents whose children were in this age range were approached with their child present, provided an explanation of the study purpose, and were invited to participate. The majority of parents and their children agreed to participate. There were 77 children between the ages of 6 and 8 (*M* = 7;7; SD = 0;10; 42 girls) and 53 children between the ages of 10 and 12 (*M* = 11;2; SD = 0;10; 33 girls). The racial and ethnic composition of the children was as follows: 74.4% White, 9.4% Biracial, 5.4% Asian, 4.7% Black or African American, 3.1% American Indian/Alaskan Native, and 3.1% Other.

#### Materials and Procedure

Children were tested individually and the overall procedure was as follows: First, children completed a mood rating (Time 1). Next, they were asked to recall a disappointing event (instructions described below) and then were asked to rate their mood again after the mood induction (Time 2). Children were then randomly assigned to one of two drawing conditions: distract or express. After drawing for 5 min, children were asked to rate their mood a final time (Time 3). Finally, children were asked some questions about the drawing activity – how absorbed they were in the activity, how much they enjoyed the activity, and how well they thought they had done drawing. The college’s institutional review board approved the study and parents provided written consent. Children ages 7 and older provided written assent and children aged 6 provided oral assent.

##### Mood induction

In order to induce a negative mood, children were instructed as follows: “I want you to think of a time when you wanted something really good to happen to you and it didn’t and you felt really upset and disappointed. I want you to close your eyes and think about how you were feeling when it didn’t happen.” Children were given up to 1 min to think of the event and were then asked to recall the event to the experimenter. The experimenter then asked children to cover their eyes with their hands (the experimenter demonstrated this to children) and focus on the event they had thought of for 30 s.

##### Activity

Children were randomly assigned to one of two conditions: distract or express. There were no differences in the gender distribution between conditions, *X*^2^ (1, *n* = 130) *p* = 0.032, 0.859, or age groups, *X*^2^ (1, *n* = 130) = 0.032, *p* = 0.858. In the distract condition, children were asked to draw a skyscraper. If needed, children were provided clarification that this was a really tall building. In the express condition, children were asked to draw the event they had thought of. All children were asked to draw for 5 min and were given a 9″ × 11″ piece of white paper and a set of colored markers.

##### Mood ratings

Children were presented with five schematic faces ranging from very sad to very happy (Rader and Hughes, 2005). Children were asked to select the face that represented how they were feeling.

##### Absorption

To rate children’s absorption in the task, children were asked the following: “While you were drawing did you forget about (the experimenter named the event) or you couldn’t stop thinking about (the experimenter named the event).” Because it might be difficult for young children to understand responses on a Likert scale, the responses presented to children followed the administration of Harter’s Self Perception Profile of Children (Harter, 1985). After children responded to this question, they were asked whether their answer choice was “sort of true” or “really true.” This resulted in four responses for the scale (1 = couldn’t stop thinking about it, really true; 2 = couldn’t stop thinking about it, sort of true; 3 = forgot about it, sort of true; and 4 = forgot about it, really true) with a higher score indicating more absorption in the activity.

##### Enjoyment and perceived competence

After drawing, children were asked to rate on a 5-point scale from *really didn’t like it* to *really liked it*: “How much did you enjoy doing this?” They were also asked to rate on a 5-point scale from *very bad* to *very good*: “How well did you think you did on this?”

##### Demographic information

Parents were asked to complete an information questionnaire asking about their child’s age, gender, race, and whether their child had taken any art lessons outside school and if so, to indicate the number of years of lesson taken.

##### Socioeconomic status

Socioeconomic status (SES) was assessed through a questionnaire (Norton et al., 2005) given to parents asking about each parent’s highest level of education. Parents were classified into one of six categories: 1 = some high school; 2 = high school graduate or GED; 3 = some college, associates, or vocational degree; 4 = college graduate; 5 = master’s degree; and 6 = doctoral degree. Children received an SES score based on the average parental score. If only one parent’s education was given, that was used as the parental score. Parent’s education can be considered a proxy for SES (Hollingshead and Redlich, 1958) and has been used in previous research.

### Results

Table 1 presents the means and standard deviations by condition and age group at Time 1, Time 2, and Time 3. The majority of children had not taken any formal art lessons outside the regular school curriculum (82.8%). Of those children that had taken any formal art lessons, the average number of years of lessons was 1.2 (SD = 0.9). On average, parents scored a 4.2 on the SES measure (SD = 1.0), which would indicate that parents had on average a bachelor’s degree.

**TABLE 1:** Means and standard deviations for mood by condition and age group at Time 1, Time 2, and Time 3.

		**Time 1**		**Time 2**		**Time 3**	
	***n***	***M***	**SD**	***M***	**SD**	***M***	**SD**
**Younger**							
Distract	38	4.3	0.8	2.3	0.9	4.2	0.7
Express	39	4.3	0.7	2.3	0.9	3.9	0.8
**Older**							
Distract	27	4.0	0.8	2.3	0.8	4.0	0.8
Express	26	4.1	0.7	2.5	0.5	3.5	0.8

#### Mood Improvement

To examine whether the conditions differed in mood improvement, a mixed design ANOVA was run with time (3) as the repeated measure and condition (2), age group (2), and gender (2) as the between subjects factors. There was an effect of time, *F*(2,244) = 243.126, MSE = 0.456, *p* < 0.001, *n*_*p*_^2^ = 0.666. Paired sample *t*-tests showed that mood decreased after the mood induction (from Time 1 to Time 2), *t*(129) = −20.863, *p* < 0.001, *d* = −1.82; and mood increased after drawing (from Time 2 to Time 3), *t*(129) = 16.985, *p* < 0.001, *d* = 1.48. There was no effect of condition, *F*(1,122) = 0.451, *p* = 0.503, *n*_*p*_^2^ = 0.004; age group, *F*(1,122) = 2.284, *p* = 0.133, *n*_*p*_^2^ = 0.018; or gender, *F*(1,122) = 0.710, *p* = 0.401, *n*_*p*_^2^ = 0.006.

There was an interaction between time and condition, *F*(2,244) = 4.268, *p* = 0.015, *n*_*p*_^2^ = 0.034. To determine the source of the interaction, an ANOVA was run separately for each time point by condition. There was no difference in mood ratings between conditions before or after the mood induction, *F*(1,128) = 0.122, *p* = 0.728, *d* = −0.07; and *F*(1,128) = 0.293, *p* = 0.589, *d* = −0.09. However, there was a difference between conditions after drawing: children in the distract condition (*M* = 4.1; SD = 0.7) had a higher mood than children in the express condition (*M* = 3.7, SD = 0.9), *F*(1,128) = 5.871, *p* = 0.017, *d* = 0.43.

There was also an interaction between time and age group, *F*(2,244) = 4.298, *p* = 0.015, *n*_*p*_^2^ = 0.034. To determine the source of the interaction, an ANOVA was run separately for each time point by age group. There was no difference in mood ratings between the groups after the mood induction, *F*(1,128) = 0.525, *p* = 0.470, *d* = −0.14. The younger group reported a higher mood rating than the older group before the mood induction, *F*(1,128) = 4.047, *p* = 0.046, *d* = 0.37; and after drawing, *F*(1,128) = 5.103, *p* = 0.026, *d* = 0.40.

There was also an interaction between time and gender, *F*(2,244) = 6.364, *p* = 0.002, *n*_*p*_^2^ = 0.050. An ANOVA was run separately for each time point by gender. Girls reported a higher mood than boys before the mood induction, *F*(1,128) = 5.461, *p* = 0.021, *d* = 0.42. There was no difference in mood ratings between girls and boys after the mood induction, *F*(1,128) = 2.874, *p* = 0.092, *d* = −0.30; or after drawing, *F*(1,128) = 2.319, *p* = 0.130, *d* = 0.27. There were no other two-way, three-way, or four-way interactions, *p*s > 0.05.

#### Absorption

**FIGURE 1:**
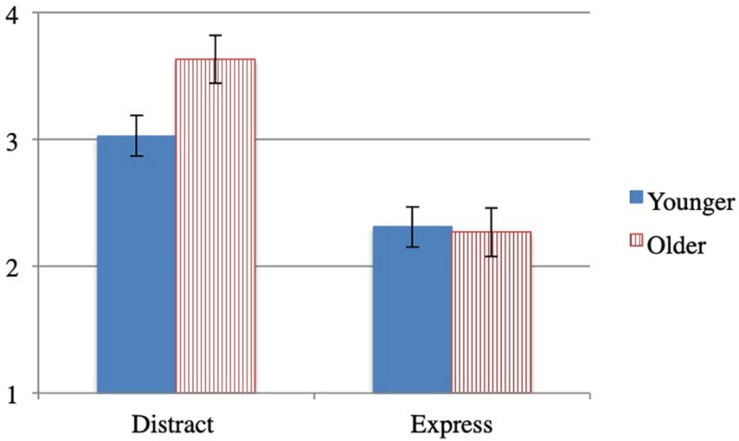
Study 1 mean absorption by condition and age group.

An univariate ANOVA by condition and age group on the absorption ratings (Figure 1), revealed an effect of condition, *F*(1,126) = 35.404, *p* < 0.001, *n*_*p*_^2^ = 0.219: children in the distract condition (*M* = 3.3, SD = 0.9) were more absorbed in drawing than children in the express condition (*M* = 2.3, SD = 1.1). There was no effect of age group *F*(1,126) = 2.613, *p* = 0.108, *n*_*p*_^2^ = 0.020. There were also no interaction between condition and age group, *F*(1,126) = 3.374, *p* = 0.069, *n*_*p*_^2^ = 0.026.

#### Enjoyment and Competence

A multivariate ANOVA by condition and age group on the enjoyment and perceived competence ratings (Table 2) revealed an effect of condition on enjoyment *F*(1,126) = 4.312, *p* = 0.040, *n*_*p*_^2^ = 0.033; but not competence, *F*(1,126) = 3.293, *p* = 0.072, *n*_*p*_^2^ = 0.025: children in the distract condition enjoyed the activity more than those in the express condition. There was an effect of age group on competence *F*(1,126) = 23.450, *p* < 0.001, *n*_*p*_^2^ = 0.157; but not enjoyment, *F*(1,126) = 2.391, *p* = 0.125, *n*_*p*_^2^ = 0.019: younger children reported higher perceived competence than older children. There was no interaction between condition and age group for enjoyment, *F*(1,126) = 0.412, *p* = 0.522, *n*_*p*_^2^ = 0.003; or competence *F*(1,126) = 1.127, *p* = 0.290, *n*_*p*_^2^ = 0.009.

**TABLE 2:** Means and standard deviations for enjoyment and perceived competence by condition and age group.

		**Enjoyment**		**Perceived competence**	
	***n***	***M***	**SD**	***M***	**SD**
**Younger**					
Distract	38	4.5	0.6	4.0	0.9
Express	39	4.1	0.9	3.9	1.0
**Older**					
Distract	27	4.2	1.0	3.3	1.1
Express	26	4.0	1.0	2.9	0.8

#### Factors Contributing to Mood Improvement

Finally, the role of absorption, enjoyment, and perceived competence in children’s mood improvement was examined. First, a mood improvement change score was calculated by subtracting Time 2 mood from Time 3 mood. Next, a regression was performed with absorption, enjoyment, and perceived competence as the independent variables and the mood improvement change score as the dependent variable. Children’s level of absorption (*B* = 0.167, *p* = 0.046) and perceived competence (*B* = 0.297, *p* = 0.002) but not enjoyment (*B* = 0.087, *p* = 0.366) predicted mood improvement.

### Conclusion

Study 1 replicated and extended prior research (Drake and Winner, 2013) by demonstrating that drawing improves mood more when used as a form distraction rather than expression even when the content of the drawing is neutral (i.e., a building). Children in the distract condition also reported higher levels of absorption and enjoyment in the drawing activity and this could account for why drawing to distract is more effective in improving mood than drawing to express. Study 2 examined the role that imagination plays in mood improvement through drawing, testing the hypothesis that drawing tasks that involve creating an imaginary world are more absorbing and thereby result in greater mood elevation.

## Study 2

### Methods

#### Participants

Children (*n* = 236) between the ages of 6 and 12 were recruited from local art and children’s museums. The same recruitment methods used in Study 1 were used here. Five children were removed from the sample because they were unable to think of a sad event (*n* = 4) or did not draw for the allotted time (*n* = 1). Thus, the final sample consisted of 231 children between 6 and 12 years old. There were 138 children between the ages of 6 and 8 (*M* = 7;5; SD = 0;11; 75 girls) and 93 children between the ages of 10 and 12 (*M* = 11;2; SD = 0;10; 54 girls). The racial and ethnic composition of the children was as follows: 31.6% White, 23.4% Black or African American, 15.6% Biracial, 14.7% Hispanic or Latino, 11.7% Asian, 2.2% Other, and 0.9% American Indian or Alaskan Native.

#### Materials and Procedure

Study 2 followed the same procedure as Study 1 except that there were two distract conditions. In the real condition, children were asked to draw a scene containing real-world elements (a dog chasing a robber). In the imagine condition, children were asked to draw a structurally similar scene containing imaginary elements (a dragon chasing a witch).

##### Activity

Children were randomly assigned to one of three conditions: real, imagine, or express. There were no differences in the gender distribution across conditions, *X*^2^ (2, *n* = 231) = 0.063, *p* = 0.969, or age groups, *X*^2^ (2, *n* = 231) = 0.283, *p* = 0.868. In the real condition, children were asked to draw a dog chasing a robber; and in the imagine condition, they were asked to draw a dragon chasing a witch. In the express condition, children were asked to draw the event they had thought of. All children were asked to draw for 5 min and were given a 9″ × 11″ piece of white paper and a set of colored markers.

### Results

Table 3 presents the means and standard deviations by condition and age group at Time 1, Time 2, and Time 3. As in Study 1, the majority of children had not taken any formal art lessons outside the regular school curriculum (83.1%). Of those children that had taken any formal art lessons, the average number of years of lessons was 1.8 (SD = 1.2). On average, parents scored a 4.0 on the SES measure (SD = 1.2), which would indicate that parents had on average a bachelor’s degree.

**TABLE 3:** Means and standard deviations for mood by condition and age group at Time 1, Time 2, and Time 3.

		**Time 1**		**Time 2**		**Time 3**	
	***n***	***M***	**SD**	***M***	**SD**	***M***	**SD**
**Younger**							
Imagine	46	4.4	0.7	2.2	1.1	4.1	0.9
Real	43	4.4	0.7	2.1	0.9	4.0	0.9
Express	49	4.3	0.7	2.4	0.9	3.7	0.9
**Older**							
Imagine	33	4.1	0.7	2.6	0.8	4.0	0.9
Real	26	4.1	0.8	2.4	0.6	3.9	0.7
Express	34	4.0	0.7	2.6	0.9	3.5	1.1

#### Mood Improvement

To examine whether the conditions differed in mood improvement, a mixed design ANOVA was run with time (3) as the repeated measure and condition (3), age group (2), and gender (2) as the between subjects factors. There was an effect of time, *F*(2,438) = 308.221, MSE = 0.669, *p* < 0.001, *n*_*p*_^2^ = 0.584. Paired sample *t*-tests showed that mood decreased after the mood induction (from Time 1 to Time 2), *t*(230) = −23.171, *p* < 0.001, *d* = −1.52; and mood increased after drawing (from Time 2 to Time 3), *t*(230) = 18.263, *p* < 0.001, *d* = 1.20. There was no effect of condition, *F*(2,219) = 1.350, *p* = 0.216, *n*_*p*_^2^ = 0.012; age group, *F*(1,219) = 0.537, *p* = 0.465, *n*_*p*_^2^ = 0.002; or gender, *F*(1,219) = 0.067, *p* = 0.795, *n*_*p*_^2^ = 0.0.

There was an interaction between time and condition, *F*(4,438) = 3.920, *p* = 0.004, *n*_*p*_^2^ = 0.034. To determine the source of the interaction, a multivariate ANOVA was run for each time point by condition. There was no difference in mood ratings across conditions before or after the mood induction, *F*(2,228) = 0.075, *p* = 0.927, *n*_*p*_^2^ = 0.001, and *F*(2,228) = 1.302, *p* = 0.274, *n*_*p*_^2^ = 0.011, respectively. However, there was a difference across conditions after drawing, *F*(2,228) = 6.635, *p* = 0.002, *n*_*p*_^2^ = 0.055. Bonferroni *post hoc* tests revealed that children in the imagine (*M* = 4.1; SD = 0.9) and real conditions (*M* = 4.0; SD = 0.8) had a higher mood rating than children in the express condition (*M* = 3.6, SD = 1.0) after drawing, *p* = 0.002 and *p* = 0.0032. There were no differences in mood ratings after drawing between the imagine and real conditions, *p* = 1.0.

There was also an interaction between time and age group, *F*(2,438) = 6.911, *p* = 0.001, *n*_*p*_^2^ = 0.031. To determine the source of the interaction, a multivariate ANOVA was run separately for each time point by age group. There was no difference in mood ratings between the age groups after drawing, *F*(1,229) = 1.115, *p* = 0.292, *n*_*p*_^2^ = 0.005. However, the younger (*M* = 4.4; SD = 0.7) group reported a higher mood rating than the older group (*M* = 4.1; SD = 0.7) before the mood induction, *F*(1,229) = 9.481, *p* = 0.002, *n*_*p*_^2^ = 0.40; and the older group (*M* = 2.5; SD = 0.8) reported a higher mood rating than the younger group (*M* = 2.3; SD = 1.0) after the mood induction, *F*(1,229) = 4.730, *p* = 0.031, *n*_*p*_^2^ = 0.20.

In contrast to Study 1, there was no interaction between time and gender, *F*(2,438) = 2.365, *p* = 0.095, *n*_*p*_^2^ = 0.011. There were also no other two-way, three-way, or four-way interactions, *p*s > 0.05.

#### Absorption

**FIGURE 2:**
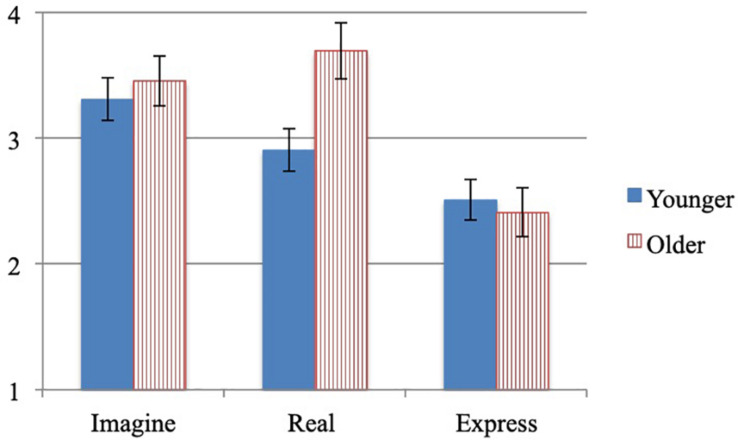
Study 2 mean absorption by condition and age group.

An univariate ANOVA by condition and age group on the absorption ratings (Figure 2), revealed an effect of condition, *F*(2,224) = 15.654, *p* < 0.001, *n*_*p*_^2^ = 0.123. Bonferroni *post hoc* tests revealed that children in the imagine (*M* = 3.4; SD = 1.0) and real conditions (*M* = 3.2; SD = 1.2) were more absorbed in the activity than children in the express condition (*M* = 2.5, SD = 1.2), both at *p* < 0.001. There were no differences in absorption ratings between the imagine and real conditions, *p* = 1.0, no effect of age group *F*(1,224) = 3.274, *p* = 0.072, *n*_*p*_^2^ = 0.014, and no interactions between condition and age group, *F*(2,224) = 2.842, *p* = 0.060, *n*_*p*_^2^ = 0.025.

#### Enjoyment and Perceived Competence

A multivariate ANOVA by condition and age group on the enjoyment and perceived competence ratings (Table 4), revealed no effect of condition on enjoyment *F*(2,225) = 0.384, *p* = 0.681, *n*_*p*_^2^ = 0.003; or competence, *F*(2,225) = 0.534, *p* = 0.587, *n*_*p*_^2^ = 0.005. There was an effect of age group on enjoyment, *F*(1,225) = 22.635, *p* <.001, *n*_*p*_^2^ = 0.091; and competence *F*(1,225) = 49.256, *p* < 0.001, *n*_*p*_^2^ = 0.180: younger children reported more enjoyment and higher perceived competence than older children. There was an interaction between condition and age group for perceived competence, *F*(2,225) = 3.837, *p* = 0.023, *n*_*p*_^2^ = 0.033; but not enjoyment *F*(2,225) = 0.658, *p* = 0.519, *n*_*p*_^2^ = 0.006. To determine the source of the interaction for perceived competence, a series of ANOVAs were run by age group separately for each condition. While the younger group reported significantly greater perceived competence than did the older group for both conditions, the differences between the age groups was greatest (as indicated by the effect size) for the real condition (*p* < 0.001, *d* = 1.20), followed by the imagine condition (*p* < 0.001, *d* = 1.05), and express conditions (*p* < 0.001, *d* = 0.50).

**TABLE 4:** Means and standard deviations for enjoyment and perceived competence by condition and age group.

		**Enjoyment**		**Perceived competence**	
	***n***	***M***	**SD**	***M***	**SD**
**Younger**					
Imagine	46	4.4	0.8	4.0	1.0
Real	43	4.6	0.7	3.9	0.9
Express	49	4.6	0.6	3.7	0.7
**Older**					
Imagine	33	4.0	1.1	2.9	1.1
Real	26	3.9	1.0	2.7	1.1
Express	34	4.1	0.9	3.3	0.9

#### Factors Contributing to Mood Improvement

As in Study 1, a regression was performed with absorption, enjoyment, and perceived competence as the independent variables and the mood improvement change score as the dependent variable. Consistent with Study 1, children’s level of absorption (*B* = 0.135, *p* = 0.029) and perceived competence (*B* = 0.301, *p* < 0.001) but not enjoyment (*B* = 0.119, *p* = 0.089) predicted mood improvement.

### Conclusion

Study 2 demonstrated that drawing improves mood when drawing real or imaginary content that is distracting and improves mood more than drawing to express. Contrary to hypothesis, entering an imaginary world did not improve mood more and did not result in greater absorption or enjoyment than did drawing something from real life. Children in both distracting conditions (real and imaginary) were more absorbed in the activity and enjoyed the activity more than children in the express condition. Study 3 examined what children spontaneously draw when upset. Do they use drawing to distract or express? What does the content of their drawings look like?

## Study 3

### Methods

#### Participants

Children (*n* = 81) between the ages of 6 and 12 were recruited from local art and children’s museums. The same recruitment methods used in Study 1 and Study 2 were used here. There were 50 children between the ages of 6 and 8 (*M* = 7;9; SD = 0;10; 27 girls) and 31 children between the ages of 10 and 12 (*M* = 11;1; SD = 0;9; 17 girls). The racial and ethnic composition of the children was as follows: 39.0% White, 26.0% Black or African American, 14.3% Biracial, 10.4% Asian, 7.8% Hispanic or Latino, 1.3% American Indian or Alaskan Native, and 1.3% Other.

#### Materials and Procedure

Study 3 followed the same procedure as Study 1 and Study 2 with two exceptions. First, children were not randomly assigned to a condition, and after the sad mood induction all children were simply instructed to draw whatever they liked for 5 min. After drawing, children responded to a series of questions about their drawing experience (outlined below).

##### Activity

**FIGURE 3:**
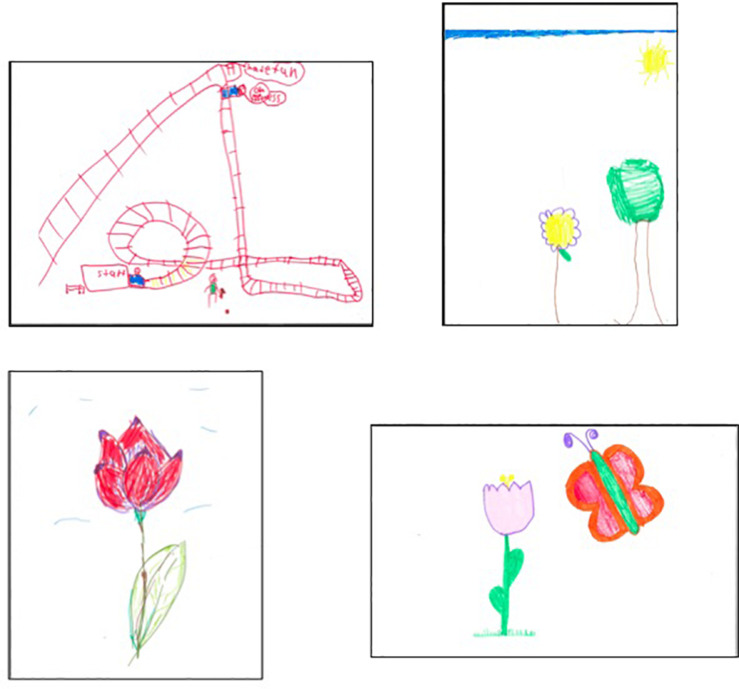
Study 3 sample of children’s drawings. Younger children drew activities they enjoyed **(top, left)** and items they felt competent drawing **(top, right)**. Older children drew items they observed **(bottom, left)** or had thought of **(bottom, right)**.

After the mood induction, children were given a 9 × 11 piece of paper and markers and asked to draw whatever they wanted for 5 min. If they completed their drawing before 5 min had passed, they were encouraged to continue by adding more details to their drawing. Figure 3 presents a sample of children’s drawings by age group.

##### Drawing Experience

After they drew and rated how they were feeling, children were asked the following questions. First, they were asked “to tell me about what you drew.” Two coders independently compared children’s responses to this question with children’s description of the sad event they recalled and coded children as either having used drawing to distract or drawing to express (*k* = 0.903). Responses that were unrelated to the sad event the child recalled were coded as distract. For example, a 10-year thought of a time when she had to take a math test, drew herself at a museum, and was classified as using drawing to distract. Responses that focused on the sad event the child recalled were coded as express. For example, a 10-year old thought of a time when her classmates were copying homework assignments, drew the event happening, and was classified as using drawing to express.

Children were then asked, “why did you choose to draw this?” Five categories of responses were noted:

• liked or enjoyed (e.g., child liked dogs)
• self-efficacy (e.g., child knew how to draw it; made child feel creative)
• happiness (e.g., made child feel happy)
• observations or thought about (e.g., child saw red flowers; child was thinking about summer)
• other (e.g., child responded they did not know or not sure).

Two coders (who were not involved in the coding development), independently coded children’s responses into one of the five categories (*k* = 0.881).

Finally, children were asked: “what were you thinking about while drawing?” Their responses to the description of the sad event they recalled and the content of their drawings were compared to determine whether their reported thoughts were distracting or expressing, and whether their thoughts were related or unrelated to their drawing or the mood induction. Two coders (who were not involved in the coding development) coded children’s responses, (*k* = 0.836) into one of these seven categories:

• distract draw: thoughts while drawing were unrelated to disappointing event but were related to the drawing itself
• distract other: thoughts while drawing were unrelated to disappointing event and were not related to the drawing being made
• express event: thoughts while drawing were about the disappointing event
• express other: thoughts while drawing were not about the disappointing event but were emotionally charged in a negative way
• express and distract: thoughts while drawing were about the disappointing event and something unrelated to the event
• nothing: thoughts while drawing were not about anything; the child responded with “nothing” or “I don’t know”
• reappraisal: thoughts while drawing reveal a reframing of the disappointing event in a positive way.

All disagreements in coding were resolved by the author who developed the coding scheme but was not involved in coding the data.

### Results

#### Preliminary Results

**FIGURE 4:**
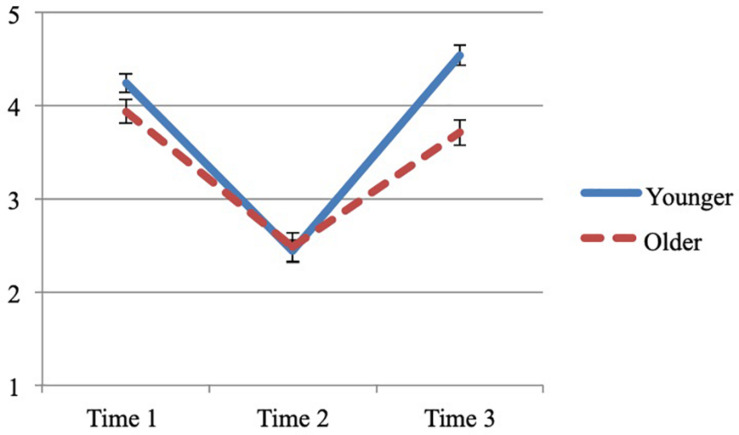
Study 3 mood improvement by time and age group.

Figure 4 presents the means and standard errors by age group at Time 1, Time 2, and Time 3. Similar to Study 1 and 2, the majority of children had not taken any formal art lessons outside the regular school curriculum (71.6%). Of those children that had taken any formal art lessons, the average number of years of lessons was 1.1 (SD = 1.0). On average, parents scored a 3.8 on the SES measure (SD = 1.3), which would indicate that parents had on average a bachelor’s degree.

### Mood Improvement

To examine whether mood improved, a mixed design ANOVA was run with time (3) as the repeated measure and age group (2) and gender (2) as the between subjects factors. There was an effect of time, *F*(2,154) = 124.025, MSE = 0.555, *p* < 0.001, *n*_*p*_^2^ = 0.617. Paired sample *t*-tests showed that mood decreased after the mood induction (from Time 1 to Time 2), *t*(80) = −13.553, *p* < 0.001, *d* = −1.50; and mood increased after drawing (from Time 2 to Time 3), *t*(80) = 14.034, *p* < 0.001, *d* = 1.55.

There was an effect of age group, *F*(1,77) = 10.517, *p* = 0.002, *n*_*p*_^2^ = 0.120. More importantly, there was an interaction between time and age group, *F*(2,154) = 6.463, *p* = 0.002, *n*_*p*_^2^ = 0.077. To determine the source of the interaction, a multivariate ANOVA was run separately for each time point by age group. There was no difference in mood ratings between age groups before or after the mood induction, *F*(1,79) = 3.595, *p* = 0.062, *n*_*p*_^2^ = 0.044; and *F*(1,79) = 0.050, *p* = 0.823, *n*_*p*_^2^ = 0.001, respectively. However, there was a difference between age groups after drawing: younger children (*M* = 4.5; SD = 0.6) had a higher mood than older children (*M* = 3.7, SD = 0.9), *F*(1,79) = 23.261, *p* < 0.001, *n*_*p*_^2^ = 0.227.

There was no effect of gender, *F*(1,77) = 0.001, *p* = 0.778, *n*_*p*_^2^ = 0.001. There was also no interaction between time and gender, time and age group, or three-way interaction between time, age group, and gender, *p*s > 0.05.

#### Absorption

An ANOVA by age group on the absorption ratings (Table 5) revealed no difference in absorption between younger (*M* = 3.3, SD = 1.1), and older children (*M* = 3.2, SD = 1.1), *F*(1,79) = 0.152, *p* = 0.698, *d* = 0.09.

**TABLE 5:** Means and standard deviations for absorption, enjoyment, and perceived competence by age group.

	**Younger (*n* = 50)**		**Older (*n* = 31)**	
	***M***	**SD**	***M***	**SD**
Absorption	3.3	1.1	3.2	1.1
Enjoyment	4.6	0.7	4.1	0.8
Perceived competence	4.0	1.0	3.0	1.0

*The highest possible score for absorption is 4.0. The highest possible score for enjoyment and perceived competence is 5.0.*

#### Enjoyment and Competence

A multivariate ANOVA by age group on the enjoyment and perceived competence ratings (Table 5) revealed an effect of age group on enjoyment, *F*(1,79) = 7.751, *p* = 0.007, *n*_*p*_^2^ = 0.089; and competence *F*(1,79) = 19.914, *p* < 0.001, *n*_*p*_^2^ = 0.201: younger children reported more enjoyment and higher perceived competence than older children.

#### Factors Contributing to Mood Improvement

As in Study 1 and Study 2, a regression was performed with absorption, enjoyment, and perceived competence as the independent variables and the mood improvement change score as the dependent variable. Contrary to Study 1 and Study 2, children’s level of absorption (*B* = 0.004, *p* = 0.907) and perceived competence (*B* = 0.053, *p* = 0.663) did not predict mood improvement. Consistent with Study 1and Study 2, enjoyment (*B* = 0.214, *p* = 0.084) also did not predict mood improvement.

#### Drawing Experience

**FIGURE 5:**
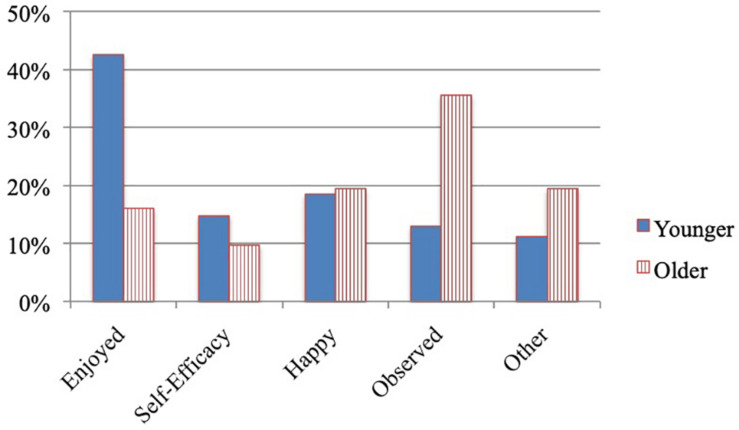
Study 3 content of children’s drawing by age group.

Regardless of age group, the majority of children used drawing as a means of distraction (93.6%) rather than as means of expression (6.4%), *X*^2^ (1, *n* = 81) = 4.194, *p* = 0.041. In terms of the content of children’s drawings (Figure 5), younger children tended to draw items they enjoyed (42.0%) and older children tended to draw items they had observed or had thought about (35.5%), *X*^2^ (4, *n* = 81) 10.819, *p* = 0.029. For example, one younger child drew roller coasters because the child “really liked roller coasters” (enjoyment) and one older child drew a red flower because the child had “seen a lot of red flowers today” (observation).

When asked about what they were thinking about while drawing, both age groups reported focusing on creating their drawing (48.1%): one 7-year old reported thinking about “the colors I was using” while a 10-year old reported thinking about “what to add to the drawing.” Children also reported thinking about distracting events unrelated to the creation of their drawing (25.9%): one 8-year old reported thinking about “animals” while a 11-year old reported thinking about “being outside with my friends.” Thus, both groups were focused on distracting thoughts unrelated to the mood induction. The remainder of the children were focused on the sad event (13.6%), nothing in particular (7.4%), something negative in tone but unrelated to the sad event (2.5%), or some form of reappraising the event (2.5%). Children’s thoughts did not differ by age group, *X*^2^ (5, *n* = 81) = 7.908, *p* = 0.161.

### Conclusion

Consistent with hypothesis, children spontaneously used drawing as a way to distract from a sad mood. Mood improved more for younger than older children, and younger children were more likely to draw things that they enjoyed, while older children were more likely to draw things that they had observed. While drawing younger and older children reported being focused on their drawing or other non-drawing distracting events.

## Discussion

It is now clear that a single session of drawing improves mood in children and does so most strongly when the act of drawing is used as a form of distraction rather than expression (Drake and Winner, 2013; Monnier et al., 2016; Brechet et al., 2020). This study sought to examine *how* drawing might elevate mood in children by examining the role of absorption, enjoyment, and perceived competence as well as entering an imaginary world play in improving mood. Finally, it examined whether children spontaneously use drawing to distract from a sad mood. Across three studies, children were asked to think of a disappointing event. Children were then randomly assigned to a drawing condition and their mood was measured before and after the mood induction and after drawing. Three main conclusions can be drawn from the studies reported here.

Study 1 replicated the finding reported by Drake and Winner (2013) that drawing improves mood when used to distract rather then express a sad mood and that the mood improvement benefits are greater for younger than older children. Unlike Drake and Winner (2013) who asked children to draw a house (which could have conjured positive or negative memories), Study 1 asked children to draw a tall building that was neutral in valence. Drawing to distract resulted in greater enjoyment and absorption than drawing to express. Consistent with work showing that younger children are less critical of their drawings than older children (Jolley, 2010), younger children reported greater perceived competence than older children. Even though engagement in drawing declines with age (Jolley et al., 2000), older children benefited from using drawing to distract but not as much as younger children. Thus, drawing to distract is an engaging and absorbing activity that can be enjoyed by children of all ages.

Study 2 found that creating content that is distracting (both the imagine and real conditions) leads to greater mood improvement and greater absorption. However, contrary to hypothesis, entering an imaginary world through drawing did not lead to greater improvement than creating real world content. Although there were no age differences in mood improvement, younger children reported more enjoyment and perceived competence than did older children, especially in the real condition. Recall that children were asked to create an imaginary scenario (dragon chasing a witch) that was structurally similar to a real scenario (dog chasing a robber). While the dragon and witch are imaginary creatures, this content was supplied to children – they did not come up with this idea on their own. It possible that differences would have been found between the imagine and real conditions, if children had simply been asked to draw something from their imagination.

Study 3 demonstrated that drawing improves mood even when children are given no instructions on what to draw. Children at both ages were more likely to use drawing to distract rather than express. Consistent with Study 1 but contrary to Study 2, there was an age effect, with younger children reporting a higher mood after drawing than older children. It possible that the content of younger children’s drawings was related to their greater mood improvement. Younger children were more likely to draw things that they enjoyed or that they felt competent drawing, while older children were more likely to draw things that they had observed or thought of (and these might not have been things they enjoyed). Finally, the majority of children reported that when drawing, they were thinking about the content of their drawings (e.g., what colors to use, what to draw next). This suggests that children were engaged, focused, and absorbed when drawing. Study 3 shows that children do not need to be given specific instructions about what to draw in order to reap the emotion regulation benefits of drawing. Children can simply be given the opportunity to draw.

Across all three studies, different findings emerged for the benefits of drawing to distract by age group. Study 1 (where children were asked to draw a skyscraper) and Study 3 (where children were asked to draw whatever they liked) showed that mood improved more for younger than older children. This finding is consistent with the work of Brechet et al. (2020) and Drake and Winner (2013) who both found that drawing to distract improved mood more for younger than older children. However, Study 2 found that drawing to distract improved mood equally for younger and older children suggesting that older children might also benefit from engaging in behavioral distraction activities such as drawing. The discrepancy across the three studies reported here might be due to differences in the content of children’s drawings. In Study 1 and Study 3, children were asked to draw something that was neutral in valence. Study 1 asked children to a draw a skyscraper and in Study 3 children were given the choice to draw whatever they wanted with the majority of children using drawing as a form of distraction. In Study 2, while the content of the distract condition drawings was intended to be neutral in valence; it did involve a potentially arousing scene of a dog (dragon) chasing a robber (witch). This arousing scene may have been less effective for younger children in improving their mood than the distraction drawing activities used in the other two studies.

Finally, the role that absorption, enjoyment, and perceived competence plays in improving mood was also examined. Similar to the findings for the developmental differences in mood improvement, an inconsistent pattern of findings emerged. In Study 1 and Study 2, children who were more engaged in the activity (as measured by absorption) and who felt they did well drawing (as measured by perceived competence) also had greater mood improvement. This was not the case for Study 3 where perceived competence and absorption did not predict mood improvement. It is possible that the findings for Study 3 are specific to a drawing activity that is freely chosen and not self-imposed. Activities that are self-selected may improve mood more than imposed activities because they are likely to lead to greater enjoyment and perceived competence (Harris, 1989). In fact, the drawing activity used in Study 3 might more closely resemble the kinds of free drawing activities that children engage in their every day lives.

### Limitations and Future Directions

This study aimed to examine the underlying factors that contribute to how drawing to distract might improve mood in children. While it was found that the drawing to distract conditions experienced more enjoyment, absorption, and perceived competence and that both absorption and perceived competence (in Study 1 and Study 2) predicted mood improvement, these measures were all completed at relatively the same time point. Children engaged in a drawing activity, rated their mood, and then completed measures of enjoyment, perceived competence, and absorption. It is unclear whether greater mood improvement resulted in greater absorption and perceived competence or vice-versa and therefore no causal claims can be made.

Another limitation of this study is that the absorption measurement was closely related to the instructions for children’s assigned drawing condition. Children in the distract condition were asked to draw something completely different from the event they recalled while children in the express condition were asked to draw and focus on that event. When completing the absorption question, children were asked whether they forgot or could not stop thinking about the disappointing event. It might not be surprising that children in the distract condition reported higher levels of absorption than children in the express condition due to the nature of the instructions for the drawing task. Finally, the findings from this study are limited to the experience children have after a mood induction. Research should examine the mood effects when children draw without any mood induction – which would align more closely with what children experience in their every day lives.

Future research would benefit from examining the benefits of drawing to distract for other emotions. For example, work with adults, has shown that drawing to distract reduces anger (Diliberto-Macaluso and Stubblefield, 2015; Genuth and Drake, 2019) and anxiety (Turturro and Drake, 2020). Finally, future research should examine the long-term benefits of drawing for children by examining whether drawing to distract or express is more beneficial. Work on drawing with adults, has shown that drawing to distract improves mood more after several days (Drake et al., 2016) and 1 month (Drake, 2019). It is unclear whether drawing on a regular basis has more of an effect on mood than just making a drawing in a single session.

## Conclusion

In sum, these three studies show that drawing to distract is an effective emotion regulation for children. When drawing to distract, children are engaging in a pleasurable and absorbing activity that allows them to adjust their sad emotions. Drawing is a universal childhood activity. It is a simple and effective tool that can be used to improve mood and well-being in children.

## Data Availability Statement

The raw data supporting the conclusions of this article will be made available by the author, without undue reservation.

## Ethics Statement

The studies involving human participants were reviewed and approved by the Boston College Institutional Review Board (for Study 1) and the CUNY Human Research Protection Program (HRPP) (for Study 2 and Study 3). Written informed consent to participate in this study was provided by the participants’ legal guardian/next of kin.

## Author Contributions

The author was responsible for all aspects of the study including the design, analysis, and write-up.

## Conflict of Interest

The author declares that the research was conducted in the absence of any commercial or financial relationships that could be construed as a potential conflict of interest.
